# Emerging In Vivo and Ex Vivo Optical Imaging Technologies in Dermatology and Dermatopathology

**DOI:** 10.3390/dermatopathology13030038

**Published:** 2026-08-20

**Authors:** Christoph Müller, Harald Kittler, Mathias Drach

**Affiliations:** Department of Dermatology, Medical University of Vienna, 1090 Vienna, Austria; christoph.mueller@meduniwien.ac.at (C.M.);

**Keywords:** reflectance confocal microscopy (RCM), line-field confocal optical coherence tomography (LC-OCT), ex vivo confocal microscopy, dermatopathology, non-invasive skin imaging, skin cancer diagnosis, digital pathology, artificial intelligence

## Abstract

Optical imaging technologies are increasingly reshaping the interface between clinical dermatology and dermatopathology. In vivo reflectance confocal microscopy (IV-RCM), line-field confocal optical coherence tomography (LC-OCT), and ex vivo confocal microscopy (EV-CM) provide tissue-level morphological information without the need for conventional histopathologic processing or with substantially reduced processing times. These technologies have enabled the concept of the “virtual biopsy” and are increasingly integrated into diagnostic workflows. However, their clinical value cannot be understood solely through conventional diagnostic performance metrics such as sensitivity and specificity. Rather, their impact depends on how the information they generate is incorporated into sequential diagnostic pathways and clinical decision-making processes. This review summarizes the principles, current applications, diagnostic performance, limitations, and future prospects of IV-RCM, LC-OCT, and EV-CM. We discuss the complementary strengths of these modalities and propose a systems perspective in which imaging technologies are viewed as components of diagnostic ecosystems linking clinical examination, dermatoscopy, pathology, surgery, and computational decision support. Emerging developments in artificial intelligence, multimodal imaging, and digital pathology are likely to further strengthen these connections and expand the role of optical imaging in dermatology and dermatopathology.

## Simple Summary

Many skin diseases, including skin cancer, are diagnosed by removing a small piece of skin and sending it to a pathology laboratory. There, the tissue is processed, stained and examined under a microscope, a process that usually takes several days. Newer instruments can show similar cellular detail much faster. Two of these techniques are placed on the skin and image the tissue in the patient. A third is used on freshly removed tissue during surgery, allowing the surgeon to assess within minutes whether the tumor has been completely removed. This review describes how the three techniques work, the conditions for which they are already used, and what their current limitations None of them replaces conventional laboratory-based pathology. We describe them as parts of an integrated process that also includes clinical examination, surgery and the pathology laboratory, and we discuss how image-analysis software may support this process in the coming years.

## 1. Introduction

The past two decades have witnessed the emergence of optical imaging technologies that extend dermatologic assessment beyond conventional clinical examination and dermatoscopy. Among these, in vivo reflectance confocal microscopy (IV-RCM), line-field confocal optical coherence tomography (LC-OCT), and ex vivo confocal microscopy (EV-CM) provide tissue-level information without immediate histopathologic processing [1,2,3,4,5,6,7]. Together, they are reshaping the interface between dermatology and dermatopathology enabling visualization of skin architecture in vivo, ex vivo, and increasingly in near real time.

Histopathology remains the diagnostic reference standard, but biopsy is invasive, time-consuming, and destructive. Optical imaging technologies seek to bridge the gap between clinical examination and pathology by providing rapid access to morphological information at cellular resolution [5,6,8,9]. The value of these technologies is often described using diagnostic performance metrics such as sensitivity, specificity, AUC, or number needed to excise (NNE). However, diagnostic performance is not an intrinsic property of the instrument alone. It emerges from the way imaging is integrated into diagnostic workflows, including patient selection, threshold setting, image interpretation, and subsequent clinical decisions. Consequently, the same technology may have markedly different clinical utility depending on where it is positioned within the diagnostic pathway and how the information it provides is used.

Emerging optical imaging technologies should therefore be viewed not simply as image-generating devices but as components of larger diagnostic ecosystems. Their contribution lies in the additional information they provide, while their clinical impact depends on the agency of clinicians and pathologists who determine when imaging is performed, how findings are interpreted, and which actions follow. This perspective becomes increasingly important as artificial intelligence (AI) is integrated with optical imaging. Although machine-learning algorithms have shown impressive performance in image analysis [10,11,12,13], their value ultimately depends on how they support clinical decision-making within real-world workflows.

The various imaging modalities occupy complementary positions within the diagnostic pathway. IV-RCM provides high-resolution en-face imaging of the epidermis and superficial dermis and is well established in the evaluation of melanocytic lesions and basal cell carcinoma [4,5]. LC-OCT extends imaging depth while providing both horizontal and vertical views that more closely resemble histopathology [3,6,7]. EV-CM facilitates rapid assessment of freshly excised tissue, supporting fast-track pathology, intraoperative consultation, and margin assessment [8,9,14,15]. Together, these technologies span the continuum from bedside imaging to digital pathology.

In this review, we examine the principles, applications, diagnostic performance, limitations, and future prospects of IV-RCM, LC-OCT, and EV-CM. Rather than considering them solely as diagnostic instruments, we discuss them as complementary components of evolving diagnostic ecosystems that connect clinical examination, dermatoscopy, imaging, pathology, surgery, and computational decision support.

## 2. In Vivo Reflectance Confocal Microscopy (IV-RCM)

### 2.1. Optical Principle and Instrumentation

IV-RCM exploits differences in refractive index between cellular and subcellular structures to generate optical contrast without exogenous staining [1,5,16]. A near-infrared laser, typically operating at approximately 785 nm, is focused onto a specific tissue plane through a high-numerical-aperture objective lens. Backscattered photons originating from the focal plane pass through a confocal pinhole, whereas out-of-focus light is rejected. This arrangement produces optical sections approximately 3–5 μm thick with a lateral resolution approaching 1 μm [16,17]. Highly refractile structures such as melanin, melanosomes, keratin, and collagen appear bright, whereas nuclei and water-rich cytoplasmic compartments appear dark. Melanin is the strongest endogenous source of contrast in human skin, making IV-RCM particularly suited for the assessment of pigmented lesions [1].

Two principal device configurations are currently used in clinical practice. Large-probe systems are attached to the skin through an adhesive ring and can acquire mosaics covering areas up to several square millimeters. Smaller handheld systems provide greater flexibility for imaging anatomically challenging sites such as periocular, intertriginous, or acral skin. Images are acquired primarily in an en-face orientation parallel to the skin surface, and vertical reconstructions require computational stacking of multiple optical sections. The resulting images differ substantially from conventional histopathology. Whereas histological sections are viewed in a vertical orientation and rely on tissue staining, IV-RCM provides horizontal optical sections based on intrinsic optical contrast. Consequently, interpretation requires dedicated training and familiarity with confocal anatomy and pathology.

### 2.2. Imaging Characteristics of Normal Skin

Understanding normal skin architecture is essential for accurate interpretation of IV-RCM images. The stratum corneum appears as bright polygonal anucleate corneocytes arranged in a characteristic surface pattern. In the granular and spinous layers, keratinocyte cell borders form the classic “honeycomb pattern,” consisting of bright cellular outlines surrounding dark nuclei [5,16]. At the dermoepidermal junction, pigmented basal keratinocytes create bright rings surrounding dermal papillae. This so-called ringed pattern represents one of the most important landmarks in confocal imaging. In the superficial dermis, collagen bundles appear as bright fibrillar structures, while capillary loops and occasionally circulating inflammatory cells can be visualized dynamically.

Many diagnostic criteria used in IV-RCM are based on deviations from these normal architectural patterns. Loss of the honeycomb architecture, disruption of papillary contours, or emergence of atypical cellular populations often provide stronger diagnostic clues than isolated morphological features.

### 2.3. Melanocytic Lesions

The diagnosis of cutaneous melanoma remains the most extensively studied indication for IV-RCM. Numerous prospective studies, systematic reviews, and meta-analyses have demonstrated that IV-RCM improves diagnostic accuracy when used as a second-level examination following dermatoscopy in clinically or dermatoscopically equivocal lesions [4,18,19,20]. Features associated with melanoma include atypical pagetoid cells, non-edged papillae, irregular junctional thickenings, atypical nests, and architectural disarray at the dermoepidermal junction [19,20,21,22]. Multiple semi-quantitative scoring systems have been developed to support melanoma diagnosis [19,21]. These approaches integrate architectural and cytological criteria into structured diagnostic frameworks. Although such algorithms improve reproducibility, they should not be viewed as substitutes for expert interpretation. Rather, they provide a framework for integrating multiple confocal observations into an overall diagnostic assessment.

IV-RCM is particularly valuable in lesions that remain equivocal after dermatoscopic evaluation. In these situations, the additional information provided by cellular-resolution imaging can substantially alter management decisions. The benefit is most evident in flat or only slightly elevated pigmented lesions where dermatoscopic asymmetry is present but the indication for immediate excision remains uncertain [4,19].

Importantly, the value of IV-RCM in melanoma diagnosis extends beyond simple lesion classification. Confocal imaging allows clinicians to evaluate the biological plausibility of competing diagnostic hypotheses. The presence of architectural disorder combined with focal cytological atypia increases confidence that a lesion represents a biologically relevant melanocytic proliferation rather than merely a dermatoscopic mimic. Thus, IV-RCM frequently contributes not by providing a definitive diagnosis, but by reducing diagnostic uncertainty.

IV-RCM has also become an important tool in the evaluation of lentigo maligna. In chronically sun-damaged skin, conventional dermatoscopic criteria are often obscured by background pigmentation and adnexal crowding. Confocal imaging permits direct visualization of atypical melanocytes surrounding follicular openings and enables more precise assessment of lesion margins before surgery [22]. This application illustrates how imaging technologies may influence not only diagnostic accuracy but also surgical planning.

### 2.4. Keratinocyte Skin Cancer

**Figure 1:**
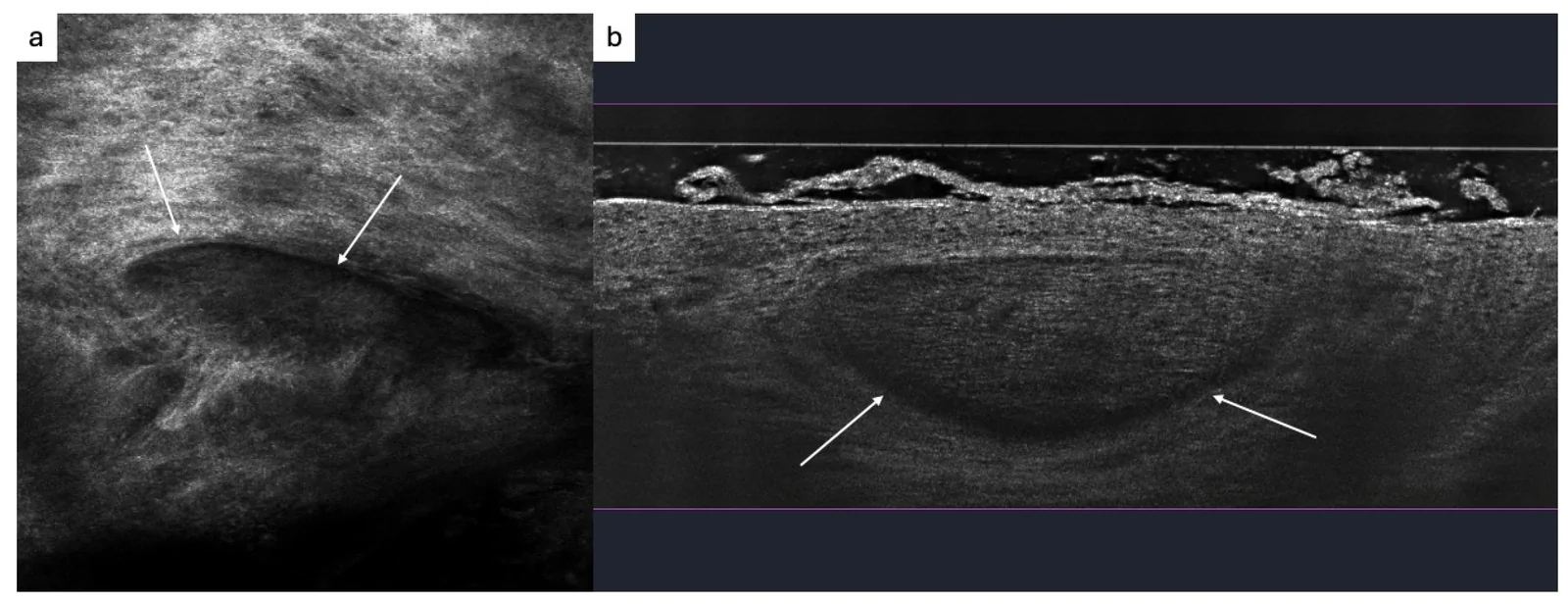
Reflectance confocal microscopy (RCM) versus line-field confocal optical coherence tomography (LC-OCT) of a basal cell carcinoma (BCC). (**a**) RCM horizontal image at the level of the superficial dermis, showing a dense, reticulated meshwork of bright collagen fibres with a central, more hyporeflective (dark) ovoid focus corresponding to a tumor island with clefting (**b**) LC-OCT vertical section (B-scan): beneath a thin, bright superficial epidermal lies a large, well-demarcated, dome-shaped hyporeflective tumor lobule within the dermis. A thin dark cleft separates the lobule from the surrounding stroma at its periphery peritumoral clefting (arrows). This figure is from Department of Dermatology, Medical University of Vienna and illustrates established RMC and LC-OCT.

Basal cell carcinoma (BCC) represents one of the most established indications for IV-RCM. Characteristic confocal features include elongated tumor islands, peripheral palisading, dark peritumoral clefts, increased vascularity, inflammatory infiltrates, and bright fibrous stromal structures (Figure 1a) [23,24].

Meta-analyses have reported sensitivities exceeding 90% and specificities approaching 90% for the diagnosis of primary BCC in selected study populations [24]. These results have established IV-RCM as one of the most accurate non-invasive imaging modalities currently available for BCC diagnosis. The technology is particularly useful in lesions located on cosmetically sensitive areas where unnecessary biopsy or treatment should be avoided. In such cases, confocal imaging may increase diagnostic confidence sufficiently to support management decisions while reducing unnecessary procedures.

IV-RCM may also contribute to BCC subtyping. Superficial and nodular tumors typically display characteristic architectural features that can be recognized with high confidence. However, infiltrative subtypes remain more challenging because diagnostically important structures often extend beyond the accessible imaging depth. This limitation is clinically relevant because treatment selection frequently depends on accurate subtype classification.

Actinic keratosis (AK) and squamous cell carcinoma (SCC) constitute another important application area. Architectural disorganization of the honeycomb pattern, atypical keratinocytes, parakeratosis, dyskeratosis, and inflammatory changes can all be visualized by IV-RCM [25]. Although limited penetration depth restricts assessment of deeply invasive SCC, the technique is well suited for evaluating epidermal atypia and treatment response.

### 2.5. Inflammatory and Infectious Dermatoses

Beyond dermato-oncology, IV-RCM has been increasingly applied to inflammatory and infectious skin diseases [26]. In psoriasis, characteristic findings include parakeratosis, elongated dermal papillae, dilated capillaries, and collections of inflammatory cells. Eczematous dermatitis may demonstrate spongiosis, exocytosis of inflammatory cells, and disruption of epidermal architecture. Lichen planus frequently exhibits interface changes and inflammatory infiltrates at the dermoepidermal junction.

Although histopathology remains the definitive diagnostic method for many inflammatory diseases, IV-RCM offers several advantages. First, it permits repeated imaging of the same lesion over time without tissue destruction. Second, it can guide biopsy site selection in clinically heterogeneous lesions. Third, it allows direct visualization of treatment-induced morphological changes.

In infectious dermatoses, IV-RCM has demonstrated particular value for the detection of ectoparasites. Demodex mites, scabies mites, and associated structures can frequently be visualized directly at the bedside [27]. This application illustrates an important principle of optical imaging: technologies may be clinically valuable even when they do not replace histopathology, provided they generate actionable information that influences patient management.

### 2.6. Therapy Monitoring and Pre-Surgical Mapping

The non-invasive nature of IV-RCM makes it particularly suitable for longitudinal monitoring. Repeated examinations can be performed without discomfort or tissue loss, allowing assessment of disease evolution and treatment response. In dermato-oncology, IV-RCM has been used to monitor non-surgical treatments such as photodynamic therapy, topical imiquimod, topical 5-fluorouracil, and targeted therapies [28]. Residual tumor may often be detected before clinical recurrence becomes apparent, potentially allowing earlier intervention.

Another important application is pre-surgical mapping. Lentigo maligna and certain BCCs frequently extend beyond clinically visible borders. IV-RCM can identify subclinical tumor extension and thereby improve surgical planning. The technology is particularly useful when tissue preservation is important, such as on the face or in anatomically complex regions.

These applications highlight an important conceptual point. The value of IV-RCM often lies less in establishing an initial diagnosis than in providing additional information at critical decision points within a diagnostic or therapeutic workflow. The same information may therefore have different clinical value depending on the context in which it is obtained.

### 2.7. Limitations of RCM

Despite its strengths, IV-RCM has several important limitations. The most fundamental limitation is penetration depth. Optical imaging is generally restricted to approximately 200–250 μm, which allows visualization of the epidermis and superficial papillary dermis but not deeper dermal structures [5,16]. A second limitation relates to image orientation. Conventional histopathology is interpreted in vertical sections, whereas IV-RCM generates horizontal optical sections. Although experienced observers can readily navigate these images, the difference creates a substantial learning curve and complicates communication between clinicians and pathologists. Third, interpretation remains operator dependent. Diagnostic performance reported in expert centers may not always be reproducible in routine clinical practice. Training requirements remain substantial, and inter-observer agreement is generally lower among inexperienced readers [25]. Finally, acquisition time, device cost, limited reimbursement, and restricted availability continue to limit dissemination. These practical considerations emphasize that technological capability alone does not determine clinical impact. Adoption depends equally on workflow integration, training infrastructure, and economic considerations.

## 3. Line-Field Confocal Optical Coherence Tomography (LC-OCT)

### 3.1. Technical Principle

Line-field confocal optical coherence tomography (LC-OCT) represents a newer generation of optical imaging technology that combines principles of optical coherence tomography and confocal microscopy [3,6,29,30]. By integrating interferometric depth gating with confocal spatial filtering, LC-OCT achieves near-cellular resolution while extending imaging depth beyond that of conventional IV-RCM. The technology employs a broadband light source and a Linnik-type interferometer equipped with high-numerical-aperture objectives. Reflected light from tissue is compared with a reference beam, allowing precise localization of structures in depth. The combination of low-coherence interferometry and confocal detection yields a spatial resolution approaching 1 μm and a penetration depth of up to approximately 500 μm in human skin [6]. This increased penetration depth represents one of the major advantages of LC-OCT. Whereas IV-RCM primarily visualizes the epidermis and superficial papillary dermis, LC-OCT frequently allows assessment of deeper dermal structures, including larger portions of keratinocyte tumors and adnexal units.

Perhaps even more important than increased depth is the ability to acquire both horizontal and vertical images in real time. Vertical sections resemble conventional histopathology more closely than IV-RCM images and are therefore often easier for histopathologists and dermatologists to interpret. Three-dimensional reconstructions can be generated rapidly, providing a comprehensive view of lesion architecture. The current generation of commercial devices also integrates dermatoscopy directly into the imaging workflow, enabling precise correlation between surface morphology and subsurface structures. This multimodal approach exemplifies the broader trend toward integration of complementary information sources within a single diagnostic platform.

### 3.2. Imaging Characteristics and Normal Skin

In vertical LC-OCT sections, the stratum corneum appears as a bright superficial band overlying the viable epidermis. Individual keratinocytes can frequently be distinguished, producing an appearance that resembles conventional hematoxylin-and-eosin sections despite the absence of tissue staining [29]. The dermoepidermal junction is visualized as a distinct interface separating epidermal and dermal compartments. Hair follicles, sebaceous glands, sweat ducts, and blood vessels are readily identified. Because images can be obtained in both horizontal and vertical planes, observers can switch between perspectives and evaluate structures from multiple orientations. This dual-view capability represents a major conceptual advance. Rather than forcing observers to infer three-dimensional architecture from a series of horizontal images, LC-OCT allows direct visualization of tissue architecture in planes that correspond more closely to conventional histopathology.

### 3.3. Clinical Applications of LC-OCT

#### 3.3.1. Benign Keratinocytic Lesions

**Figure 2:**
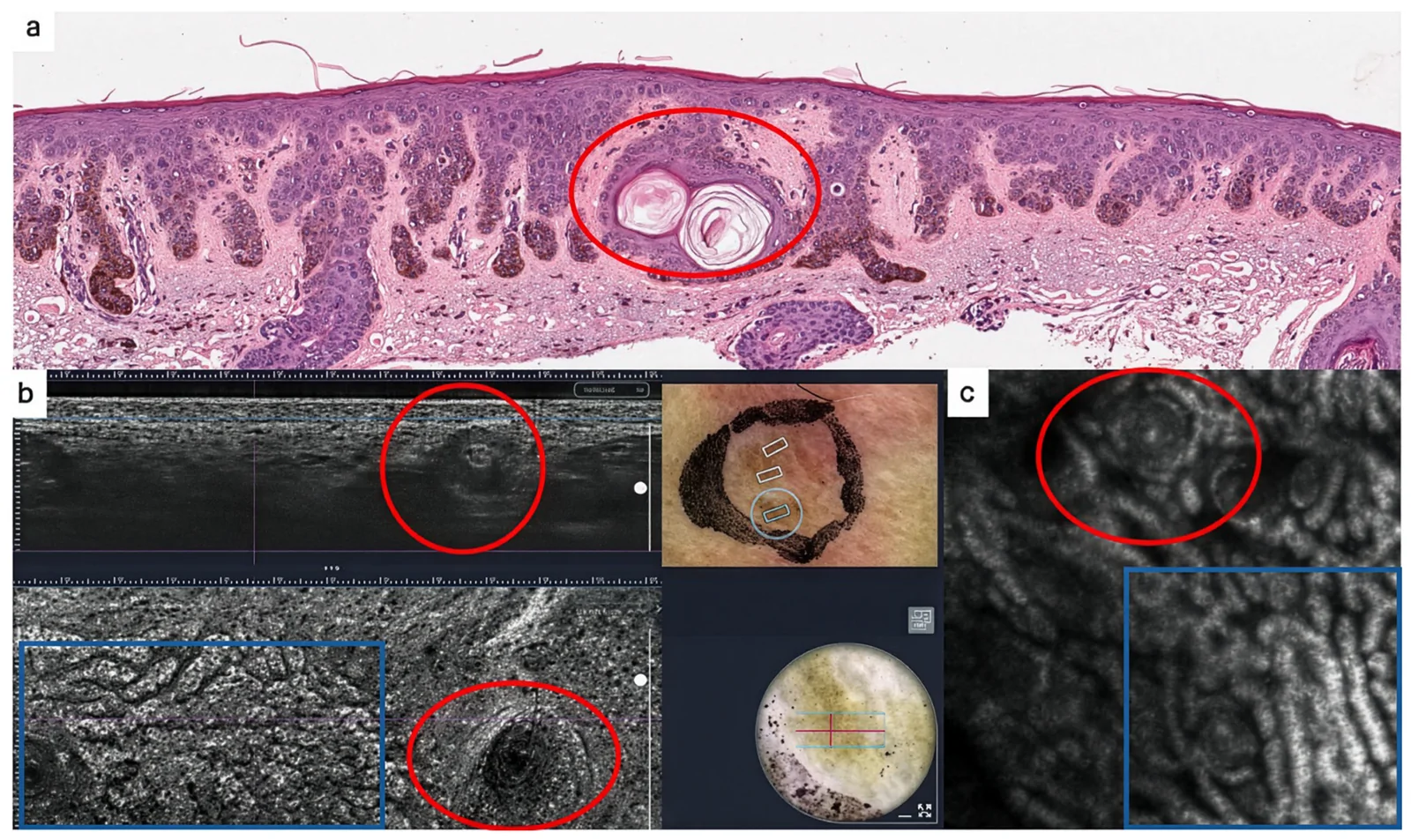
Multimodal imaging of a seborrheic keratosis. (**a**) Histopathology (haematoxylin & eosin) of the lesion; the red circle highlights a dilated, keratin-filled infundibulum/horn pseudocyst. (**b**) LC-OCT vertical section (B-scan, top) and horizontal section (bottom) of the same lesion; the blue rectangle indicates the en-face region of interest and the red circle marks milia-like cysts with onion-like appearance, blue squares indicate elongated cords of the rete ridges (**c**) RCM en-face image demonstrating similar features seen in the LC-OCT horizontal section. This figure is from Department of Dermatology, Medical University of Vienna and illustrates established multimodal imaging findings.

Seborrheic keratoses and solar lentigines are among the most common benign lesions encountered in routine dermatologic practice and frequently represent diagnostic challenges because they may mimic melanoma or BCC [31,32,33,34]. LC-OCT allows visualization of characteristic architectural features that facilitate recognition of these lesions. Seborrheic keratoses typically demonstrate papillomatous epidermal proliferation, keratin-filled invaginations, horn cysts, and sharply demarcated lesion borders (Figure 2) [31]. The vertical orientation of LC-OCT is particularly useful for appreciating these architectural patterns because it resembles the perspective familiar from conventional histopathology. Solar lentigines are characterized by elongated rete ridges, increased basal pigmentation, and preservation of normal tissue architecture [33,34]. In contrast to lentigo maligna, atypical melanocytes and significant architectural disorder are absent.

#### 3.3.2. Basal Cell Carcinoma

BCC represents the best validated indication for LC-OCT. Characteristic findings include tumor lobules, peripheral palisading, stromal clefting, and disruption of normal dermal architecture [35,36,37]. The vertical orientation of LC-OCT provides a substantial advantage over IV-RCM in this context. Tumor depth, downward proliferation, and relationships between tumor islands and surrounding tissue can be appreciated directly (Figure 1b). These features are particularly relevant when treatment decisions depend on subtype classification or assessment of tumor extent. Several prospective studies have reported sensitivities approaching 95–99% and specificities ranging from 85–95% for BCC diagnosis [35,36,37]. These results compare favorably with dermatoscopy alone and establish LC-OCT as one of the most accurate non-invasive modalities currently available for BCC. The ability to estimate tumor depth and subtype may be particularly valuable when considering non-surgical treatments or when planning tissue-sparing surgery. In such situations, LC-OCT provides information that extends beyond simple lesion classification and directly influences therapeutic decision-making.

#### 3.3.3. Actinic Keratosis and Squamous Cell Carcinoma

LC-OCT has also demonstrated promising performance in actinic keratosis and squamous cell carcinoma [38,39]. Architectural disorganization, atypical keratinocytes, dyskeratosis, and disruption of the dermoepidermal junction can be visualized with high resolution. Particularly noteworthy is the development of the PRO score (Proliferation, Round cells, Origin of disarrangement), which was specifically designed for grading actinic keratosis and assessing progression along the actinic keratosis–squamous cell carcinoma continuum [38,39]. Such scoring systems may improve standardization and facilitate longitudinal monitoring. The greater penetration depth of LC-OCT compared with IV-RCM is especially relevant in keratinocyte tumors because clinically important structures frequently extend into deeper portions of the epidermis and superficial dermis.

#### 3.3.4. Melanocytic Lesions

The evidence base for LC-OCT in melanocytic lesions is less mature than that for RCM [40,41]. Recent studies have demonstrated the ability of LC-OCT to visualize pagetoid cells, atypical melanocytes, junctional nests, dermal nests, and architectural disorder. One of the most attractive aspects of LC-OCT in melanocytic lesions is its ability to provide both horizontal and vertical views. Horizontal sections facilitate comparison with RCM, whereas vertical sections allow direct correlation with conventional histopathology. Although current evidence suggests promising diagnostic performance, standardized diagnostic criteria remain under development [40,41].

#### 3.3.5. Inflammatory and Infectious Disorders

LC-OCT has also been applied to inflammatory skin diseases, including psoriasis, eczema, lichen planus, and interface dermatitis [42]. Characteristic features such as spongiosis, inflammatory infiltrates, and epidermal architectural changes can be visualized with excellent histopathological correlation. The increased penetration depth of LC-OCT may provide particular advantages in diseases involving adnexal structures or deeper portions of hair follicles. This characteristic differentiates LC-OCT from IV-RCM and expands the range of disorders that can be evaluated non-invasively. In infectious dermatoses, LC-OCT has demonstrated utility in the bedside evaluation of selected parasitic and superficial infectious conditions [42].

### 3.4. Pre-Surgical Mapping and AI-Assisted Imaging

**Figure 3:**
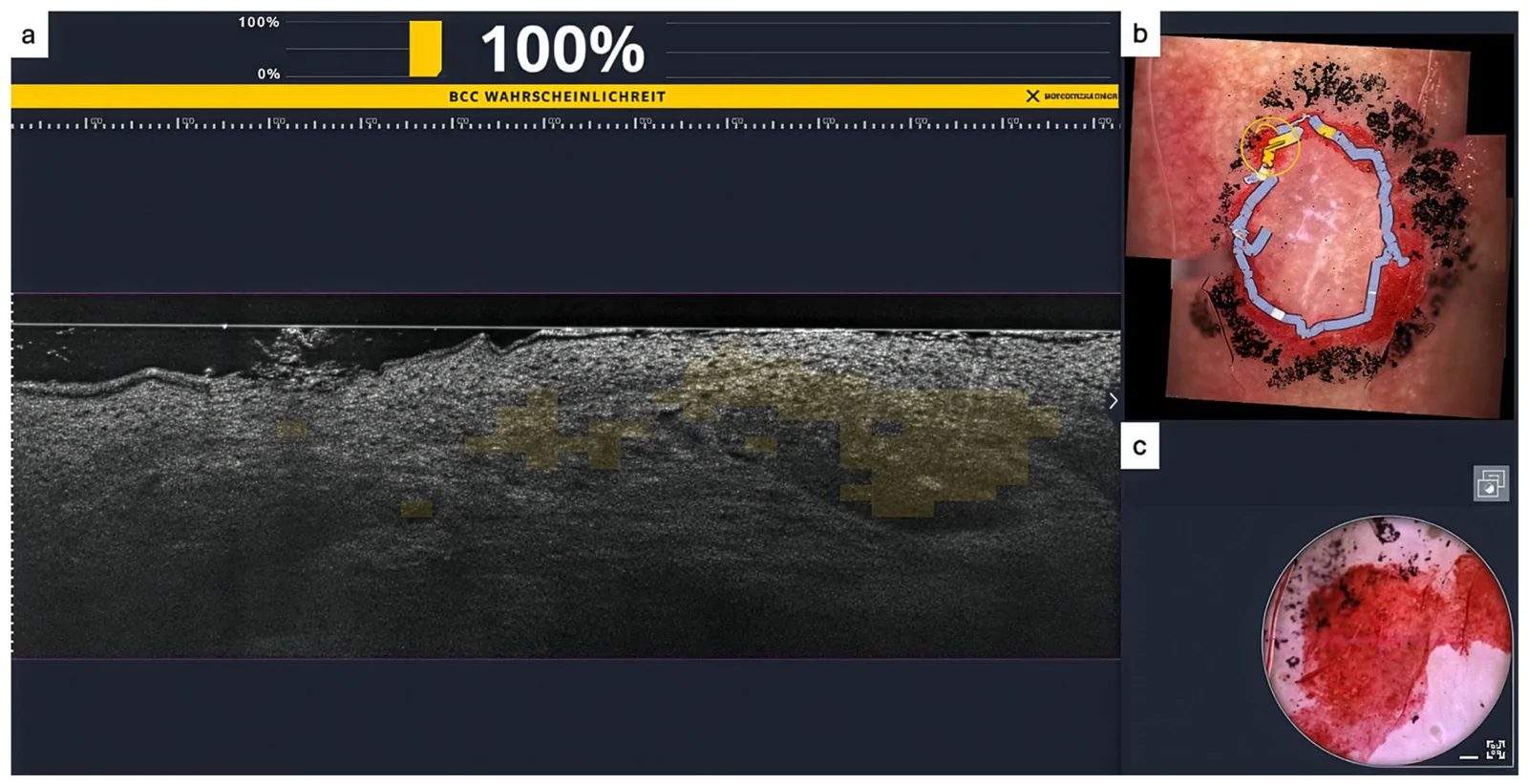
AI-assisted LC-OCT and pre-surgical margin assessment in BCC. (**a**) LC-OCT vertical section (B-scan) with the device’s integrated deep-learning classifier output overlaid on the acquisition interface; the on-screen readout indicates a BCC probability of 100%, and the regions classified as suspicious for BCC are highlighted in yellow. (**b**) Corresponding dermoscopic image of the lesion with the LC-OCT–guided tumor outline and planned excision margin delineated: yellow lines indicate areas classified as BCC-suspicious by the AI, and blue lines indicate tumor-free margins. (**c**) Dermoscopic image of the same lesion at higher magnification. This figure is from Department of Dermatology, Medical University of Vienna and illustrates established multimodal imaging findings with LC-OCT and AI-support.

One of the most promising emerging applications of LC-OCT is pre-surgical mapping of keratinocyte skin cancer, particularly BCC [10,11]. High-resolution three-dimensional imaging allows delineation of subclinical tumor extension and may improve surgical planning, particularly in cosmetically sensitive areas. Recent systems have integrated AI directly into the acquisition workflow. Deep-learning algorithms can identify tumor-suspicious regions, generate lesion probability scores, and highlight areas of interest in real time (Figure 3) [10]. Importantly, the clinical value of such systems does not arise solely from improved classification performance. Their potential lies equally in reducing variability between readers, facilitating training, and improving consistency across institutions.

### 3.5. Limitations of LC-OCT

Despite its advantages, LC-OCT has important limitations. Although penetration depth exceeds that of RCM, imaging remains restricted to approximately 500 μm and therefore cannot fully replace histopathology in deeply invasive tumors [6,29]. The technology is also relatively new. Diagnostic criteria for keratinocyte tumors are increasingly well established, but standardized algorithms for melanoma, inflammatory disease, and adnexal pathology remain under development [38,40,42]. Device costs, training requirements, and limited availability continue to restrict adoption. Furthermore, despite impressive image quality, interpretation remains observer dependent and requires substantial expertise. These limitations highlight a recurring theme in optical imaging: technological capability alone is insufficient to generate clinical value. Training, workflow integration, standardization, and appropriate patient selection remain equally important determinants of success.

## 4. Ex Vivo Confocal Microscopy

### 4.1. Concept and Position Within the Diagnostic Pathway

EV-CM occupies a unique position among optical imaging technologies because it serves as a bridge between clinical imaging and conventional pathology [8,9]. Whereas IV-RCM and LC-OCT image tissue directly on the patient, EV-CM is performed on freshly excised tissue specimens outside the body. Conceptually, EV-CM addresses a different clinical problem than in vivo imaging. Rather than helping clinicians decide whether tissue should be removed, it helps determine what has been removed and whether further intervention is necessary. In modern dermatopathology and dermatosurgery, three principal approaches are currently used for rapid tissue assessment: conventional frozen sections, stimulated Raman-based approaches, and EV-CM [43,44,45]. Among these, EV-CM offers a unique combination of speed, image quality, and operational simplicity. The technique enables generation of digital images that closely resemble conventional hematoxylin-and-eosin sections while requiring only minimal tissue preparation. Consequently, EV-CM has increasingly been described as a form of “digital fast pathology” rather than merely another imaging modality [8].

### 4.2. Principle and Instrumentation

**Figure 4:**
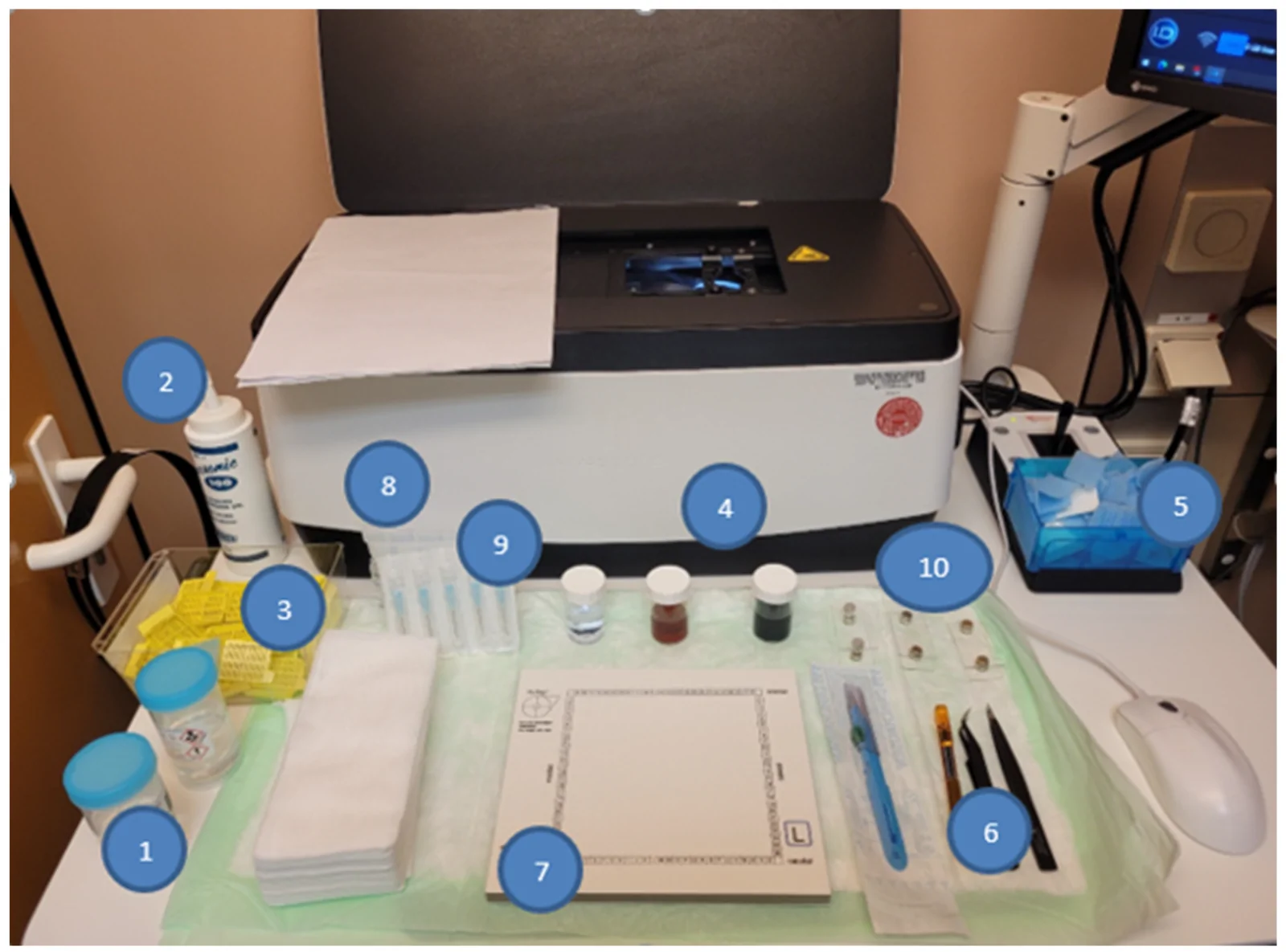
Working station of a EV-CM. (1) buffered formalin, (2) ultrasound gel, (3) histology cassettes, (4) staining solutions: alcohol (70%), acridin orange and fast green, (5) sponges, (6) tweezers and knife, (7) cardboard plate, (8) saline solution 0.9%, (9) needles, (10) microscope slides with magnets. This figure is from Department of Dermatology, Medical University of Vienna and illustrates the EV-MC workplace.

**Figure 5:**
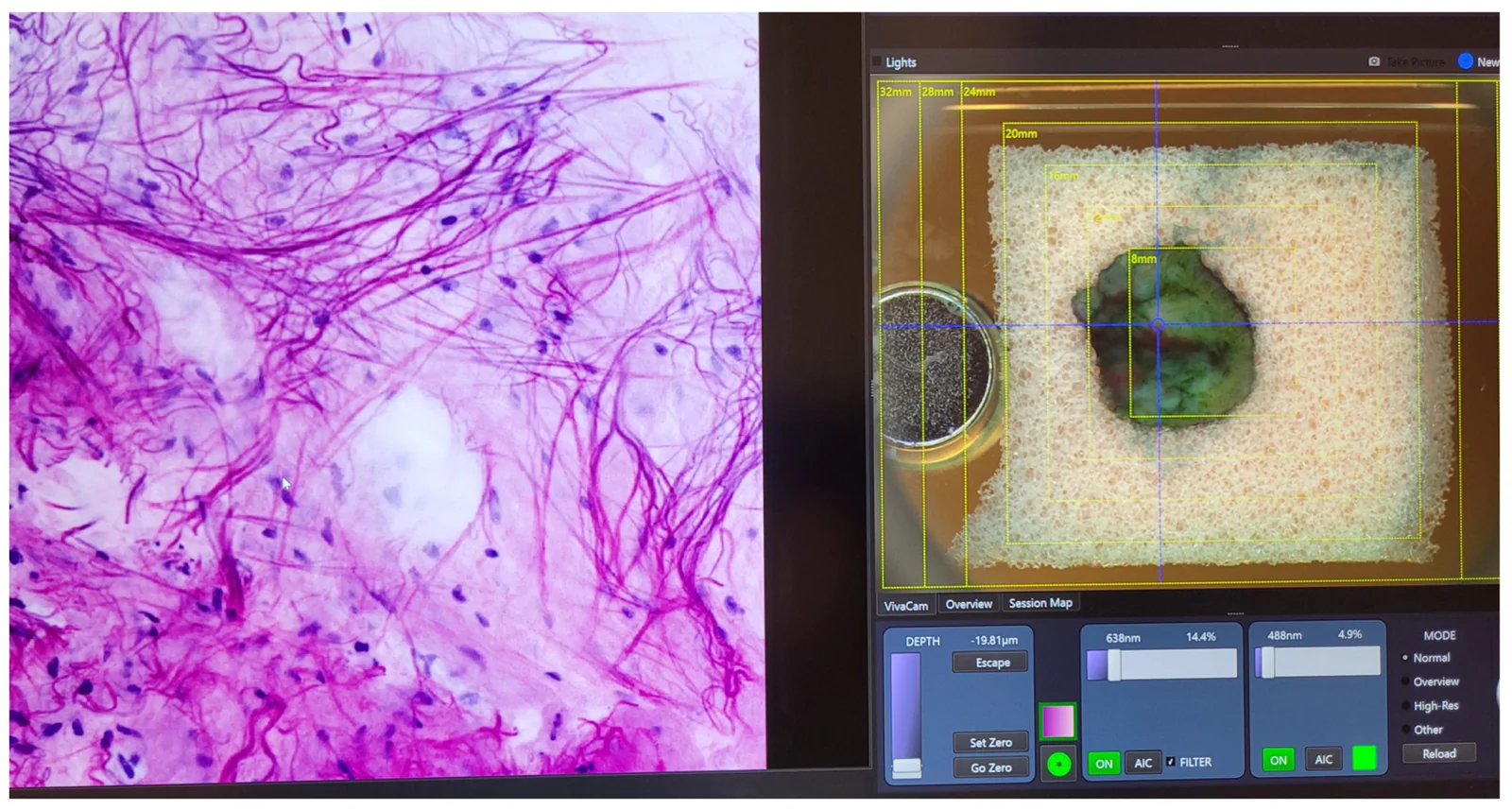
Screen shot of scanning procedure with the EV-CM: The focus is set prior every run with focusing at histologic structures. This figure is from Department of Dermatology, Medical University of Vienna and illustrates established ex vivo confocal images and the scanning procedure.

Freshly excised tissue specimens are briefly fixed and stained using fluorescent dyes such as acridine orange and fast green [8,46]. Following preparation, specimens are mounted on glass slides and positioned within the imaging system. The instrument typically utilizes dual laser wavelengths and combines reflectance and fluorescence signals to generate composite images resembling conventional histopathology [8]. The resulting images can be examined at high magnification, digitally navigated, and interpreted immediately after acquisition. The entire workflow generally requires between five and fifteen minutes, substantially shorter than conventional histopathologic processing [8,47]. From an operational perspective, one of the most attractive aspects of EV-CM is its relative simplicity. The technology can often be operated by a single individual and requires only limited laboratory infrastructure. This facilitates deployment in surgical environments where rapid turnaround times are essential (Figure 4 and Figure 5)

### 4.3. Imaging Characteristics

Unlike IV-RCM and LC-OCT, which rely exclusively on endogenous contrast, EV-CM employs fluorescent staining to enhance visualization of cellular structures. As a result, image appearance more closely resembles conventional histopathology. Epidermal architecture, adnexal structures, tumor islands, inflammatory infiltrates, and stromal changes can all be recognized with considerable fidelity. For many observers, interpretation is therefore more intuitive than interpretation of in vivo confocal images. The ability to generate digital H&E-like images creates a natural interface between clinical dermatologists, surgeons, and pathologists. This characteristic may ultimately prove as important as technical performance because it facilitates communication across disciplines.

### 4.4. Clinical Applications of Ex Vivo Confocal Microscopy

#### 4.4.1. Margin Assessment in Dermatosurgery

**Figure 6:**
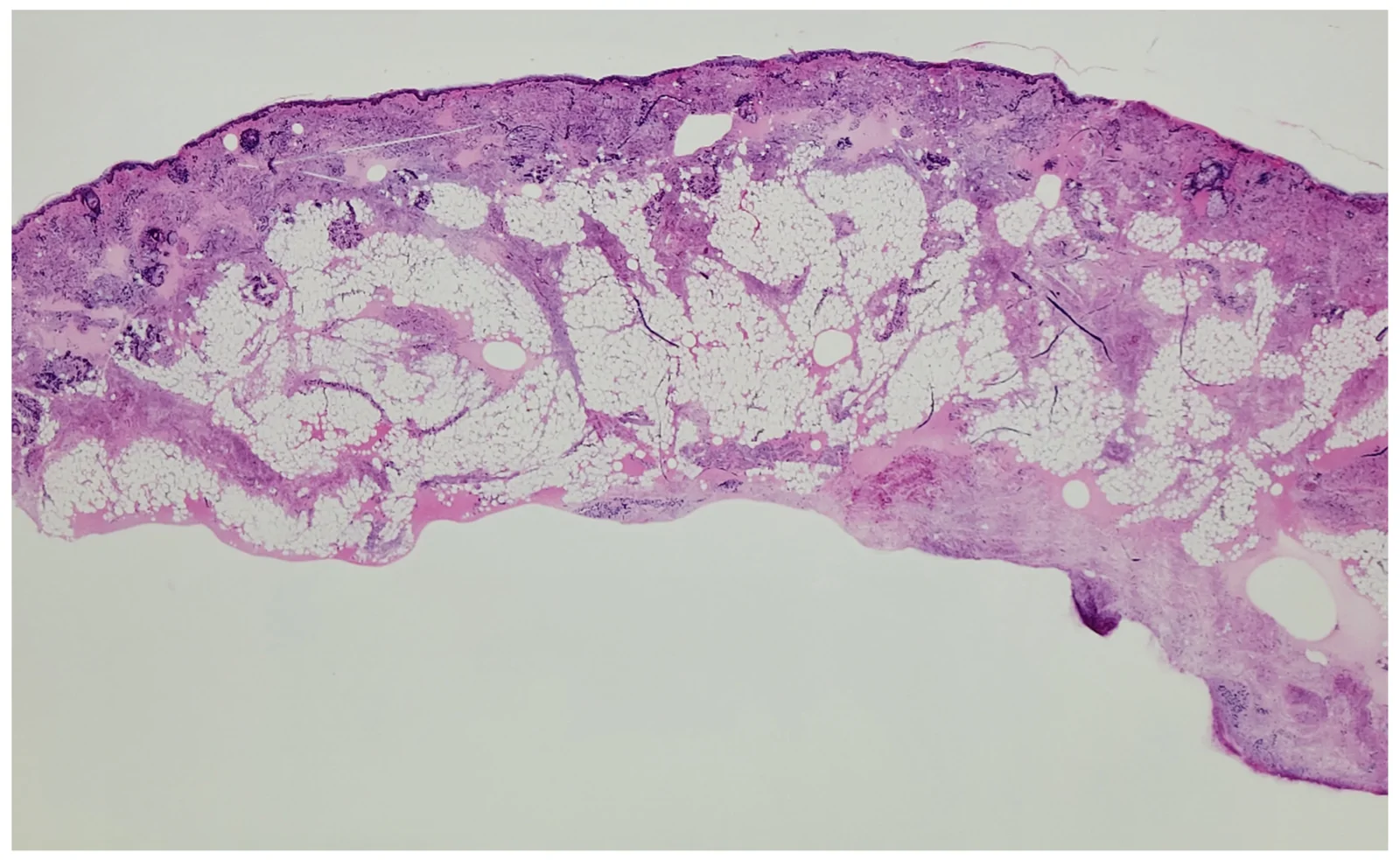
Overview of a finished scan showing precisely the entire structures of the skin. This figure is from Department of Dermatology, Medical University of Vienna and illustrates established ex vivo confocal images.

**Figure 7:**
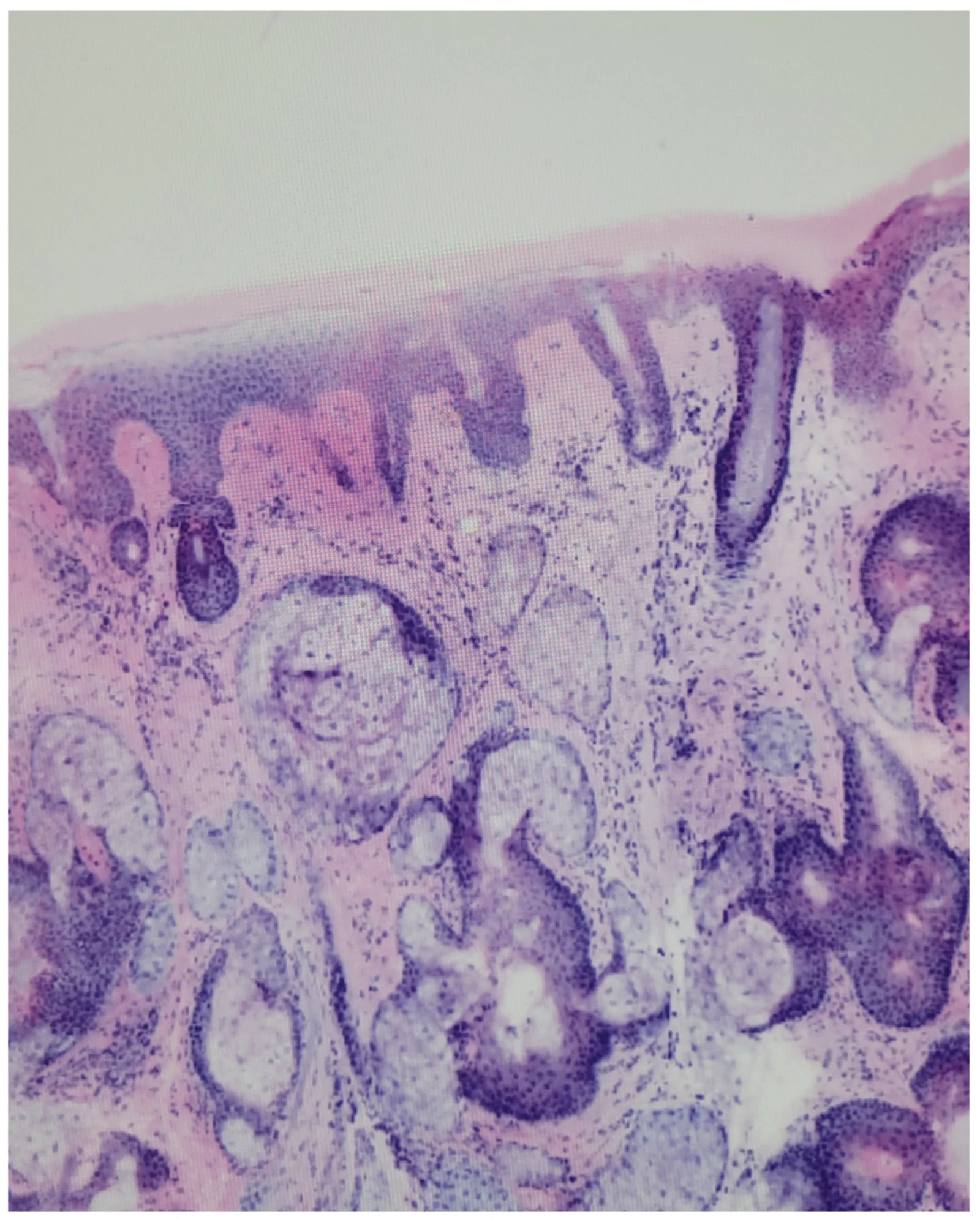
Detailed picture of the upper dermis and epidermis. This figure is from Department of Dermatology, Medical University of Vienna and illustrates established ex vivo confocal images.

The most established application of EV-CM is intraoperative margin assessment during dermatosurgical procedures [8,9,14,45]. In this setting, freshly excised tissue can be examined within minutes, allowing rapid identification of residual tumor at surgical margins. This application is particularly relevant for BCC, where complete tumor removal must be balanced against preservation of healthy tissue. Conventional frozen sections remain the reference standard for intraoperative assessment in many centers, but EV-CM offers substantially shorter processing times while preserving tissue for subsequent conventional histopathological evaluation if required [44]. Importantly, the clinical value of EV-CM in this setting is not simply its diagnostic accuracy. Rather, its value emerges from its position within the surgical workflow. Information obtained within minutes can influence immediate surgical decisions, whereas equally accurate information delivered hours or days later may have substantially less clinical impact (Figure 6 and Figure 7).

#### 4.4.2. Basal Cell Carcinoma

BCC remains the most extensively studied indication for EV-CM [8,15,45,46]. Characteristic tumor islands, peripheral palisading, stromal clefting, and associated stromal reactions can be readily identified. Several studies have demonstrated excellent concordance between EV-CM and conventional histopathology for the assessment of surgical margins [14,45]. As a result, the technique has increasingly been adopted in specialized dermatosurgical centers. Particularly attractive is the ability to examine large tissue surfaces rapidly. Whereas conventional histopathology samples only selected sections, EV-CM allows evaluation of substantially larger areas of tissue. This may improve detection of focal residual tumor and facilitate more comprehensive margin assessment.

#### 4.4.3. Melanocytic Lesions and Melanoma

Melanoma represents a more challenging application because interpretation requires recognition of subtle cytological and architectural abnormalities [48,49]. Nevertheless, studies have demonstrated that EV-CM can identify characteristic features of melanocytic proliferations and provide accurate estimates of tumor thickness [48,49]. Although conventional histopathology remains essential for definitive melanoma diagnosis, EV-CM may contribute to rapid triage, margin assessment, and correlation between clinical imaging and pathology. Particularly interesting is the possibility of creating a continuous imaging pathway from dermatoscopy to IV-RCM or LC-OCT and finally to EV-CM and conventional histopathology. Such workflows may facilitate clinicopathological correlation and improve understanding of how imaging findings relate to tissue architecture.

#### 4.4.4. Inflammatory and Autoimmune Diseases

Applications of EV-CM extend beyond oncology. In autoimmune blistering diseases such as bullous pemphigoid, ex vivo confocal approaches have demonstrated promising results and may complement or potentially simplify conventional diagnostic workflows [50]. Similarly, rare inflammatory dermatoses and selected inflammatory infiltrates can be evaluated using EV-CM [51]. Although these applications remain less established than oncologic uses, they highlight the versatility of the technology.

#### 4.4.5. Beyond Dermatopathology

EV-CM is not restricted to dermatology. Applications have been reported in gastrointestinal pathology, liver pathology, neurosurgery, and other surgical disciplines [43,47,52,53]. This broader adoption suggests that EV-CM addresses a general challenge in pathology: the need to generate high-quality morphological information rapidly enough to influence clinical decisions. The success of the technology in multiple specialties reinforces the concept that imaging should be understood not merely as visualization, but as a mechanism for accelerating information flow within clinical workflows.

### 4.5. Diagnostic Accuracy

Reported diagnostic performance varies according to indication and study design. Systematic reviews have generally demonstrated sensitivities and specificities exceeding 80%, with concordance rates frequently surpassing 90% when compared with conventional histopathology [14]. For margin assessment of keratinocyte skin cancer, several studies have reported particularly favorable results [15,45]. These findings suggest that EV-CM can provide clinically actionable information with a level of accuracy sufficient for many intraoperative applications. However, diagnostic accuracy must be interpreted in context. Performance estimates obtained in specialized centers may not necessarily generalize to all settings.

### 4.6. Advantages and Limitations

Several features distinguish EV-CM from both conventional histopathology and in vivo imaging techniques. First, turnaround time is remarkably short. Diagnostic-quality images can frequently be obtained within 5–15 min, enabling near real-time pathological assessment [8,46]. Second, the technique generates digital images that resemble conventional H&E sections, facilitating interpretation and communication among clinicians and pathologists. Third, operational requirements are relatively modest. Tissue preparation is simple, laboratory infrastructure requirements are limited, and workflows can often be managed by a single operator. Fourth, tissue is preserved. Unlike frozen sections, EV-CM does not consume substantial portions of the specimen and allows subsequent conventional histopathological examination if required.

Despite these advantages, important limitations remain. The size of tissue specimens that can be examined in a single acquisition remains restricted. Larger specimens frequently require multiple scans and image stitching [46]. Interpretation still requires specialized training. Although images resemble histopathology, confocal artifacts and differences in staining characteristics create potential sources of error. Furthermore, conventional histopathology remains necessary in many situations, particularly when ancillary techniques such as immunohistochemistry or molecular testing are required. Finally, widespread implementation depends on economic considerations, training infrastructure, and integration into existing pathology workflows.

## 5. Integration in Clinical Workflows and Role of AI

The preceding sections have focused on individual technologies. However, IV-RCM, LC-OCT, and EV-CM are best understood not as standalone diagnostic tests but as components of larger diagnostic workflows. Diagnostic performance is not an intrinsic property of an instrument; it emerges from the interaction between technology, observer, patient selection, diagnostic thresholds, and clinical objectives. In routine practice, imaging modalities are used sequentially. Clinical examination and dermatoscopy may be followed by IV-RCM or LC-OCT for equivocal lesions, while EV-CM can support rapid assessment of excised tissue before conventional histopathology. The value of each modality therefore depends not only on the information it provides but also on the information already available and the decisions that remain to be made. Dermatoscopy, IV-RCM, LC-OCT, EV-CM and conventional pathology should thus be regarded as complementary rather than competing technologies. Together they span the continuum from lesion detection to pathological evaluation, forming a diagnostic ecosystem.

The convergence of optical imaging, digital pathology, and artificial intelligence (AI) is one of the most important developments in contemporary dermatology and dermatopathology. Deep-learning models have shown promising performance in the interpretation of IV-RCM and LC-OCT images and are already being incorporated into commercial imaging platforms [10,11,12,13]. However, the future role of AI extends beyond lesion classification. AI may assist with image annotation, quantification of morphological features, structured reporting, and integration of information across multiple modalities, including dermatoscopy, confocal imaging, and pathology. In particular, EV-CM may provide a natural bridge between in vivo imaging and digital pathology because of its close resemblance to conventional histology. As with imaging technologies themselves, the clinical value of AI depends on how it is embedded within diagnostic workflows. The greatest impact is therefore likely to come not from incremental gains in classification accuracy but from improved integration, interpretation, and communication of information across the diagnostic pathway.

Although IV-RCM, LC-OCT, and EV-CM share the goal of tissue-level imaging, their optimal indications and practical requirements differ, and these differences determine when each modality should be used (Table 1). IV-RCM is well studied for melanocytic lesions and lentigo maligna. LC-OCT adds vertical, histology-like sections that are particularly advantageous for keratinocyte cancers and for estimating tumor depth and subtype. EV-CM is primarily an intraoperative and fast-pathology tool for margin assessment.

For basal cell carcinoma, these modalities can support lesion characterisation, subtype differentiation, margin assessment, therapeutic planning and response monitoring [8,24,35,54]. Practical implementation is influenced by several factors like the learning curve, equipment availability and cost, which vary between academic centres and community practice. Cost-effectiveness data suggest that if imaging is used to triage clinically equivocal lesions, the resulting reduction in unnecessary excisions can compensate for device and training costs, but formal health-economic evidence remains limited [4,55]. For all three modalities the clearest benefit is seen at specific decision points: monitoring treatment response, guiding intraoperative margin assessment and avoiding biopsies that would not change management.

The reliability of these techniques also depends on the reproducibility of image interpretation. Inter-observer agreement is generally fair to good for well-defined criteria and among experienced readers but decreases for subtle findings and less experienced observers, underscoring the need for standardized interpretation criteria and structured reporting [25,56]. Consensus terminologies, representative image glossaries and scoring systems improve consistency and support training. These are currently most established for IV-RCM while comparable standardization efforts for LC-OCT and EV-CM are still under way [38,56].

In a multicenter reader study, a majority diagnosis by nine readers reached a sensitivity of 100% compared with a mean of 88.9% for individual readers, with a nearly unchanged specificity, suggesting that confocal images contain more diagnostic information than a single reader extracts [56]. Structured and potentially AI-supported reading may therefore improve reproducibility across centers.

Finally, several challenges must be addressed before these technologies can be adopted at scale. Regulatory pathways differ by region and device, and only some systems have obtained CE marking or FDA clearance for specific indications, which limits routine clinical use. Integration with digital pathology and laboratory-information systems, including standardized image formats, storage and reporting, will be essential for embedding optical imaging, and EV-CM in particular, into existing dermatopathology workflows. Most importantly, much of the current evidence originates from specialised centres and retrospective designs. Prospective, multicentre validation studies with standardized protocols and patient-centred endpoints are needed to confirm generalisability and to define the settings in which each modality provides the greatest benefit.

## 6. Future Directions and Conclusions

Future developments will likely focus on three areas. First, ongoing technical advances will continue to improve image quality, acquisition speed, penetration depth, three-dimensional visualization, and ease of use. Second, multimodal workflows integrating complementary imaging modalities will become increasingly important. Beyond IV-RCM, LC-OCT, and EV-CM this will likely include techniques not covered in this review, such as OCT angiography, high-frequency ultrasound, and spectroscopic methods including Raman spectroscopy and electrical impedance spectroscopy. By combining different modalities, these approaches may provide a more comprehensive characterization of skin lesions than any single modality alone. Third, artificial intelligence is expected to evolve from image classification toward workflow support, including lesion selection, image interpretation, margin assessment, image–pathology correlation, and integration of information across multiple diagnostic modalities. At the same time, increasing adoption will require standardized terminology, reporting systems, and training curricula. Future research should place greater emphasis on patient-centered outcomes, including reductions in unnecessary procedures, improved surgical outcomes, shorter diagnostic delays, and cost-effectiveness. Ultimately, the future of optical imaging will depend not only on better instruments but also on better integration of information across diagnostic systems. Importantly, clinical benefit arises not from information alone but from its translation into action. Future studies should therefore evaluate complete diagnostic pathways and patient-centered outcomes, such as reductions in unnecessary biopsies, improved surgical planning, shorter diagnostic delays, and improved patient outcomes, rather than focusing exclusively on isolated measures of diagnostic accuracy.

## Figures and Tables

**Table 1:** Comparison of the characteristic imaging features of in vivo reflectance confocal microscopy (IV-RCM), line-field confocal optical coherence tomography (LC-OCT), and ex vivo confocal microscopy (EV-CM) across the principal skin conditions and clinical indications discussed in this review. DEJ, dermoepidermal junction; H&E, hematoxylin and eosin; AK, actinic keratosis; SCC, squamous cell carcinoma.

Indication	IV-RCM	LC-OCT	EV-CM
Normal skin	Honeycomb epidermis, ringed (edged) papillae, bright collagen; en-face acquisition, vertical views only by computational stacking	Layered epidermis with distinct DEJ on vertical (H&E-like) and horizontal views; adnexa and vessels resolved	H&E-like architecture of excised tissue after fluorescent staining
Melanocytic lesions/melanoma	Pagetoid cells, irregular junctional thickenings, non-edged papillae, atypical nests, junctional disarray	Pagetoid cells, atypical melanocytes, junctional and dermal nests, architectural disorder	Cytological and architectural atypia; tumor-thickness estimation and margins; histology still required for definitive diagnosis
Basal cell carcinoma	Tumor islands, peripheral palisading, dark clefts, increased vascularity, bright stroma	Tumor lobules, palisading, stromal clefting; vertical view shows depth and subtype	Tumor islands, palisading, clefting on fresh excisions
Actinic keratosis/SCC	Honeycomb disarray, atypical keratinocytes, parakeratosis, dyskeratosis (limited depth)	Architectural disarray, atypical keratinocytes and dyskeratosis at greater depth	Keratinocyte atypia on excised specimens (limited published experience)
Seborrheic keratosis/solar lentigo	Corded and cerebriform pattern with keratin in seborrheic keratosis; preserved ringed pattern in solar lentigo; en face	Papillomatosis, horn and milia-like cysts, keratin invaginations, elongated rete (vertical view)	Papillomatous architecture on excised tissue (rarely required)
Inflammatory dermatoses	Spongiosis, exocytosis, interface changes, dilated capillaries	Same features visualized to greater depth, including adnexal structures	Selected applications (e.g., bullous pemphigoid) on biopsy tissue
Infectious/parasitic	Direct bedside visualization of Demodex and scabies mites	Selected parasitic and superficial infectious conditions at bedside	Not a primary indication
Margin assessment/surgical planning	Pre-surgical mapping of subclinical extension, particularly in lentigo maligna	Pre-surgical mapping of subclinical tumor extension, including AI-assisted delineation	Core indication: rapid (5–15 min), tissue-preserving margin assessment of fresh excisions

## Data Availability

The figures presented in this review are intended for illustrative purposes only. They exemplify previously established findings and are not used to support the inference of any new conclusions. No new data were created or analyzed in this study. Data sharing is not applicable to this article.
